# High Genetic Diversity and Fine-Scale Spatial Structure in the Marine Flagellate *Oxyrrhis marina* (Dinophyceae) Uncovered by Microsatellite Loci

**DOI:** 10.1371/journal.pone.0015557

**Published:** 2010-12-23

**Authors:** Chris D. Lowe, David J. S. Montagnes, Laura E. Martin, Phillip C. Watts

**Affiliations:** School of Biological Sciences, University of Liverpool, Liverpool, United Kingdom; Loewe Biodiversity and Climate Research Centre, Germany

## Abstract

Free-living marine protists are often assumed to be broadly distributed and genetically homogeneous on large spatial scales. However, an increasing application of highly polymorphic genetic markers (*e.g.*, microsatellites) has provided evidence for high genetic diversity and population structuring on small spatial scales in many free-living protists. Here we characterise a panel of new microsatellite markers for the common marine flagellate *Oxyrrhis marina*. Nine microsatellite loci were used to assess genotypic diversity at two spatial scales by genotyping 200 isolates of *O. marina* from 6 broad geographic regions around Great Britain and Ireland; in one region, a single 2 km shore line was sampled intensively to assess fine-scale genetic diversity. Microsatellite loci resolved between 1–6 and 7–23 distinct alleles per region in the least and most variable loci respectively, with corresponding variation in expected heterozygosities (*H_e_*) of 0.00–0.30 and 0.81–0.93. Across the dataset, genotypic diversity was high with 183 genotypes detected from 200 isolates. Bayesian analysis of population structure supported two model populations. One population was distributed across all sampled regions; the other was confined to the intensively sampled shore, and thus two distinct populations co-occurred at this site. Whilst model-based analysis inferred a single UK-wide population, pairwise regional *F_ST_* values indicated weak to moderate population sub-division (0.01–0.12), but no clear correlation between spatial and genetic distance was evident. Data presented in this study highlight extensive genetic diversity for *O. marina*; however, it remains a substantial challenge to uncover the mechanisms that drive genetic diversity in free-living microorganisms.

## Introduction

Free-living marine protists commonly have broad distributions [1], [2], [3] and potentially large population sizes [3]. In combination with a general assertion that marine environments offer few physical barriers to dispersal this has often lead to the assumption that protist populations may be genetically homogeneous over large spatial distances [4]. This idea is largely consistent with studies based on the spatial distribution of sequence variation, typically at ribosomal gene loci, which often provide evidence for widely distributed phylotypes [e.g. [5],[6]]. However, such studies also commonly highlight extensive genetic diversity within existing morphospecies, and thus also lend support to the counter argument that free-living protists are typically spatially restricted or endemic [7]. Most recently protist biogeographers have begun to recognise that the debate has been overly generalised and largely associated with differences in opinion in defining the appropriate evolutionary unit (e.g. species-and if so under which concept, population, phylotype, or other genetic variant) to scrutinise [8]. Indeed, the increasing application of highly polymorphic genetic markers (such as microsatellites) is a recognition that defining population structure and population level genetic variation is critical to understanding the demographic processes that drive free-living protists distributions. Such high resolution studies are now revealing extensive genetic diversity and significant population structuring within free-living protist species occurring on small spatial scales on the order of tens to hundreds of kilometres [9], [10], [11].

A further reason to limit generalisations about the distributions and genetic diversity of free-living protists is the diverse array of life history strategies displayed by such organisms [8]. Varying levels of ploidy, differing frequencies of sex and recombination, and cyst formation all potentially complicate analyses of population structure in many protist taxa. For example, asexual reproduction raises the potential for rapid changes in the genetic composition of nominal populations as a result of the clonal proliferation of one or few genotypes. Such asexual proliferation and domination of a population by one or few clone(s) can potentially mask underlying patterns of diversity and population structure [12] (though note such patterns are most extreme in parasitic species). For many species, however, we have a poor understanding of life history strategies and remain ignorant of the relative frequencies of asexual reproduction and sex and subsequently the extent of clonal diversity. In a broader context, this poor characterisation has important consequences; for example, from an experimental or modelling standpoint, it is not clear whether a particular protist isolate is representative of the population from which it was derived.

To date, most population-genetic studies of marine protists have focussed on a relatively small number of ecologically or economically important species such as harmful algae, coccolithophores and diatoms [e.g. [9],[11],[13],[14],[15], [ 16], [17]]. However, the distributions of other free-living protist taxa are also of interest. Indeed, in the context highlighted above comparative studies of population genetic structure in species with contrasting life histories are likely to be highly informative. For example, the heterotrophic marine flagellate *Oxyrrhis marina* (Alveolata: Dinophyceae) is an ideal model to study population structure/dynamics and evolutionary processes in free-living marine protists as it is common in coastal habitats [18], widely-distributed [19], [20], at times ecologically relevant [21], and has a long history of use as an experimental organism [22]. Our phylogeographic studies indicate that *O. marina* is genetically differentiated on broad spatial scales [19], [23] and have established *O. marina* as a useful model for the study of biogeographic processes within free-living marine protists [e.g. 20]. Notably, an analysis of sequence variation at two gene loci (regions of cytochrome *c* oxidase I, COX1, and 5.8S internal transcribed spacer rDNA, 5.8S-ITS) indicated contrasting patterns of genetic diversity among broad geographic regions; in particular most samples isolated from coastal waters of the Northwest Atlantic were identical at these loci [19]. Such patterns of phylogeographic uniformity potentially indicate broad dispersal and limited population structuring, although insufficient sampling and limited genetic marker resolution may have contributed to an apparent lack of spatial genetic structure. In addition, observations of sex in experimental cultures of *Oxyrrhis* are poorly documented [24], and there are no estimates of the likely frequency of sex in natural populations. Consequently, an assessment of population structure requires intensive sampling and the use of highly polymorphic genetic markers.

Here, we develop the first panel of polymorphic microsatellite markers for *O. marina* and use these to (1) compare the level of genetic diversity at microsatellite loci with previous studies based on sequence variation of common phylogenetic markers and (2) assess the extent of genetic structure at two spatial scales, first on a fine-scale (∼1–2 km), within a single shore and second at a regional scale (∼1,000 km), among a range of coastal areas of Great Britain and Ireland. We uncover high levels of genetic diversity and, for the first time in this taxon, detect fine-scale spatial genetic structure. These results have clear implications for researchers using *O. marina* as an experimental model [see 22] and discussion.

## Methods

### Sample collection

**Figure 1:**
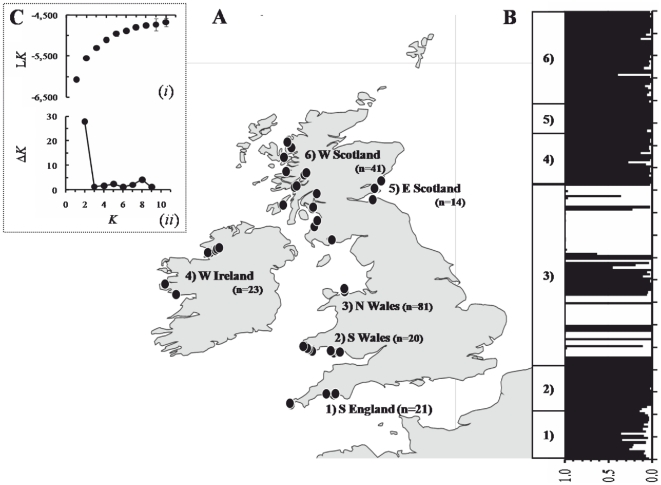
The distribution and genetic structure of *Oxyrrhis marina* sampled across UK coastal waters. **A**: The distribution of *O. marina* samples collected from UK coastal waters. Samples were grouped into 6 regions for pairwise comparisons; *n* = number of isolates per region. **B**: The probabilities of membership of individual *O. marina* isolates to hypothetical clusters in a two cluster simulation. Each bar represents an individual isolate and the proportion of the bar that is black or white represents the proportion of assignment to cluster 1 or 2 respectively. **C**: (*i*) Mean L(*K*) (±95% CI) over 5 independent runs for each *K* value; (*ii*) Ä*K*, the second order rate of change of Ln P(D) with respect to *K*; the modal value of this distribution corresponds to the true value of *K* or the uppermost level of genetic structure (see Methods and [31] for further details).

For genotyping, 200 *Oxyrrhis marina* samples were isolated from 15–100 ml of seawater obtained from coastal, predominately intertidal, habitats by the authors or collaborators (see Acknowledgements). Samples were located at six general regions around Great Britain and Ireland: (1) southern England (*n* = 21), (2) southern Wales (*n* = 20), (3) northern Wales (*n* = 81), (4) western Ireland (*n* = 23), (5) western Scotland (*n* = 41), and (6) eastern Scotland (*n* = 14), with the emphasis at the site in northern Wales (Figure 1) to make a comparison between the pattern of genetic diversity at local and regional-scales.

Clonal isolates were obtained following methods outlined in [19]. Briefly, seawater samples were inoculated with the flagellate *Dunaliella primolecta* CCAP11/34 as food; after 1–5 days, in samples where *O. marina* was visible, monoclonal cultures (hereafter termed isolates) were established. Isolates were on-grown for 1–2 weeks in 50 ml volumes (with low concentrations of *D. primolecta*); subsequently samples were pelleted by centrifugation and then stored at −20°C. DNA for genotyping was extracted using a high-salt method [25]. To provide sufficient genetic material for microsatellite library construction, 1 L cultures of two isolates (44_PLY01 and 44_ABE01 - isolated from Plymouth, UK and Aberystwyth, UK, respectively – see [19] for strain details) were established (with *D. primolecta*) and treated with penicillin/streptomycin (100 µgml^−1^) and gentamycin (50 µgml^−1^) to minimise bacterial growth. Cultures were maintained in the dark for ∼2 weeks (to prevent growth of *D. primolecta*) or until *O. marina* grazed the prey to extinction. Absence of *D. primolecta* was confirmed by PCR using universal eukaryote 18/28S rDNA primers [19]. Final *O. marina* cell concentrations were ∼5×10^4^ ml^−1^; cultures were then harvested by centrifugation, and DNA was extracted using a phenol:chloroform method [25].

### Microsatellite loci development and genotyping

To construct the partial genomic library, 40 units of *Sau*3AI (Boehringer-Mannheim) were used to digest 8–10 ìg of genomic DNA (pooled from the two isolates detailed above). Digested DNA was subsequently ligated to 50 pmol of phosphorylated linkers (S61 5′-GGCCAGAGACCCCAAGCTTCG-3′ annealed to S62 5′-PO4-GATCCGAAGCTTGGGGTCTCTGGCC-3′ [26]). DNA fragments between 500 and 1,500 bp were excised from a 1.8% agarose gel and purified using a QIAquick gel extraction kit (Qiagen). Full details of the enrichment procedure (based on [27]) are provided elsewhere [28]; briefly, we hybridized the DNA fragments with M2-80 streptavadin coated magnetic beads (Dynal) that had been incubated with 3′-biotin-labelled (CA)_12_ and (TCAC)_6_ oligonucleotides (MWG Biotech). After a series of differential stringency washes in 2× SSC and 1× SSC, the enriched DNA was made double stranded and amplified in a 25 ìl PCR (75 mm Tris-HCl, 20 mm (NH)_4_SO_4_, 0.01% (v/v) Tween 20, 0.2 mm each dNTP, 1.5 mm MgCl_2_, 25 pmol primer S61 and 1.25 units of *Taq* polymerase (ABgene). The thermal profile of the PCR was: 95°C 5 min, 25–30× [95°C 50 s, 56°C 1 min, 72°C 2 min], 72°C 10 min. The DNA was purified using a QIAquick PCR purification kit (Qiagen), ligated into pGEM®-T vector (Promega) and transformed into JM109 *E. coli* competent cells (Promega). Recombinant clones were identified using black/white screening on S-gal (Sigma) agar/ampicillin plates. Plasmids containing an insert with a microsatellite were identified by two or more amplified products after PCR primed with 50 pmol SP1 and 25 pmol of (nonbiotinylated) microsatellite oligonucleotide (either (CA)_12_ or (TCAC)_6_ see [27]). Positive clones were cycle sequenced using Big Dye™ chemistry (Applied Biosystems) and electrophoresis on an ABI3130*xl* (Applied Biosystems). Primers flanking microsatellite regions were designed using Primer3 software [29].

Microsatellite alleles were amplified by PCR in a 10 ìl reaction volume on a Dyad DNA Engine (MJ Research Inc.). PCR conditions were 95°C for 1 min, 5× [95°C 30 s, *T_a_*°C 45 s, 72°C 45 s], 25× [92°C 30 s, *T_a_*°C 45 s, 72°C 55 s], 72°C 10 min; *T_a_* is the locus-specific annealing temperature (Table 1). Each PCR contained 75 mM Tris-HCl (pH 8.8), 20 mM (NH_4_)_2_SO_4_, 0.01% (v/v) Tween 20, 0.2 mM each dNTP, 2.0 mM MgCl_2_, 5–50 ng template DNA, 3 pmol each primer, and 0.25 u *Taq* polymerase (ABgene). PCR fragments were fluorescently labelled with either 6-FAM, NED, PET or VIC fluorescent dyes (Applied Biosystems). PCR products were pooled with a 500 bp (LIZ) size standard (Applied Biosystems), separated by capillary electrophoresis on an ABI3130*xl* and sized using genemapper software (Applied Biosystems). Error rates were assessed by genotyping (*i.e.* PCR, electrophoresis, and allele scoring) a subsample of 48 individuals twice. The occurrence of null alleles and systematic scoring errors was assessed using Microchecker [30].

**Table 1:** Summary of locus characteristics, PCR details and levels of variability for nine polymorphic microsatellite loci isolated from the marine flagellate *Oxyrrhis marina* from coastal waters of Great Britain and Ireland (*n* = 200).

Locus GenBank	Dye	Primer sequence (5′ ->3′)	Repeat motif	*T_a_*	Size	*N_a_*
Om01	6-FAM	CCTCGTCCATTCAGTCTCG	(CA)_31_	53	129–166	12
GU811649		GCGAGAATACAGTCCGTAAGC				
Om03	NED	GTGCGTGATTGTCCTCAGC	(CA)_4_…(CAA)_2_…(CA)_7_	53	149–182	12
GU811650		GGAAGGAGGGTCACATAGTCC				
Om06	PET	CGCCGCTGTAGTCTTTTCC	(ACC)_3_G(CA)_6_C(CA)_7_	53	193–256	28
GU811651		AAGCGATGAATACACACTGTCG				
Om09	VIC	TGTTGCGAGTCGTTATCTTCC	(CAA)_44_	56	243–356	36
GU811652		CACAGGATGGTCCTCACAGC				
Om12	PET	GGTGTAGTACAGGGGTGTTGG	(CA)_26_	56	133–145	6
GU811653		CGTTGCCTCTAGCTTAGTTGG				
Om02	VIC	GTCGTACTCGTTCGTCAGGAG	(CA)_9_…(CA)_8_…(CA)_10_	58	200–242	20
GU811654		TGGACTGTGGAGTGATGATTG	…(CA)_10_(TG)_3_…(CA)_8_			
Om07	NED	GGTCCGTAGGTCTTTGACAGG	(CA)_32_	56	218–264	19
GU811655		ATGCTTTTGCCCATGTTGC				
Om08	PET	TTAAGCTAGCACACCAACACG	(CA)_32_	54	212–232	6
GU811656		TTCCTCCTTTTCACCTTTTGC				
Om10	6-FAM	CGAAGGATATACAACGAGAAGG	(CA)_5_…(CA)_32_	56	170–250	36
GU811657		CATATTTGCCGGTTAGTGTGG				

*T_a_*, annealing temperature of primers during PCR; Size, size range of alleles (in base pairs); *N_a_*, number of alleles.

### Genetic data analyses

Basic measures of genetic diversity - the number of alleles (*N_a_*), expected heterozygosity (*H_e_*), Wright's [31] inbreeding coefficient *f* (estimated using the method of [32]), and genotype frequencies - were calculated, using fstat v.2.9.3.2 [33], for the broad geographic regions indicated in Figure 1; the significance of any departures from expected Hardy-Weinberg equilibrium (HWE) conditions was assessed by permuting alleles among individuals within regions (1,000 permutations). genepop software (v.3.1c, http://wbiomed.curtin.edu.au/genepop/; [34]) was used to calculate the level of linkage disequilibrium between all pairs of loci within regions. Sequential Bonferroni corrections for *k* multiple tests were applied where appropriate [35].

Spatial genetic structure was assessed using the model-based clustering approach implemented by structure v. 2.3.1 (see [36] for full background) that simultaneously identifies clusters (populations) and assigns individuals to clusters using a Bayesian approach (Figure 1). Briefly, structure models *K* populations that are characterised by a set of allele frequencies at each locus. Individuals are (probabilistically) assigned to a population (or populations if they are admixed) on the basis of their multilocus genotypes, assuming unlinked loci and HWE conditions within populations. Typically, the number of distinct populations (*K*) is estimated from the model of *K* that maximises the probability of the data (in structure terminology returns the maximal value of *ln* P(D)), but for complex population structures a modal value of *ln* P(D) may not be apparent. In such instances the value of *K* at the beginning of a ‘plateau’ of estimates of *ln* P(D) may be selected as this is the smallest value of *K* that captures the major structure of the data set[37]. We also used an alternative method to detect the number of clusters by calculating the *ad hoc* measure: *ΔK* (the second order rate of change of *ln* P(D) with respect to *K*; [38]), where the modal value of *ΔK* corresponds to the most pronounced partition of the data set. Five independent runs of structure were carried out for the total data set for *K* = 1 to *K* = 10 using the admixture model and correlated allele frequencies [36]. All model runs were based on 300,000 iterations after an initial burn-in of 30,000 iterations.

fstat was used to calculate the level of genetic differentiation - *F_ST_* [32] - between the six regions, with the significance of deviation of *F_ST_* estimates from zero assessed through 2,000 permutations of genotypes between populations. Isolation by distance (IBD) genetic structure was investigated by spatial autocorrelation [39] whereby we calculated the variation in average kinship (*F_ij_*; [40]) among individual isolates separated by a range of increasing spatial scales, using spagedi version 1.2 [41]. Separate analyses were made for each of the two model clusters identified by structure (defined in the *Results* below; see also Figure 1B) as isolates belonging to one cluster are restricted to N Wales. Average (over all loci) correlograms are presented to avoid variation in correlogram profiles based on the frequencies of individual alleles that are subject to stochastic processes. Standard errors (and thus 95% confidence intervals) for average *F_ij_* were generated by jackknifing over loci.

## Results

From the enriched partial genomic library, we identified ∼300 recombinant clones (*i.e*. JM109 cells with plasmids) bearing putative microsatellite repeats, of which 171 were sequenced. PCR primers could be designed around 42 microsatellite loci; of these 25 suffered from severe stutter banding, 5 were monomorphic, and 12 were polymorphic. Nine polymorphic microsatellite loci (see Table 1 for primer details) generated reproducible genotypes and were used for genotyping; error rates per locus ranged between 0.96% and 2.88%. One marker (Om07) had a conspicuously higher error rate (5.76%), predominately as a result of a relatively high level of sample drop out for this locus.

These nine loci resolved between 1–6 and 7–23 distinct alleles per region in the least (Om08, Om12) and most (Om09, Om10) variable loci, respectively, with corresponding variation in expected heterozygosities (*H_e_*) of 0.00–0.30 and 0.81–0.93 (Table 2). Wright's [31] *f* was typically high and positive (with the exception of the three loci Om03, Om08, and Om12), with significant (*P*>0.05, *k* = 6) heterozygote deficits identified in 24 instances (*i.e.* locus per region), notably in the samples from North Wales and Eastern Scotland and all regional sample groups at the locus Om06 (Table 2). One striking feature of these data was the high level of genotypic diversity (183 different genotypes), and thus no single genotype (potential clone) dominated any of the regional samples. Matching samples occurred predominantly in the more intensively-sampled beach at North Wales, where 6 pairs of isolates had identical profiles across all 18 alleles (with each pair having a different genotype). Other apparently identical isolates likely represented chance matches rather than clonal genotypes, as they represented pairs of isolates from distant areas and all of these matches were samples that had one or more non-amplifying alleles.

**Table 2:** Basic measures of genetic diversity: expected heterozygosity (*H_e_*), Wright's [24] inbreeding coefficient (*f*) and numbers of alleles (*N_a_*) for samples of *O. marina* from six geographic regions in UK coastal waters.

Locus		S England	S Wales	N Wales	W Ireland	E Scotland	W Scotland
Om01	*H_e_*	0.860	0.864	0.727	0.827	0.672	0.865
	*f*	−0.026	0.391*	0.607*	0.300	0.554	0.331*
	*N_a_*	9	9	9	6	5	10
Om03	*H_e_*	0.573	0.609	0.761	0.626	0.439	0.544
	*f*	−0.164	−0.149	0.043	−0.110	−0.226	−0.057
	*N_a_*	7	6	9	7	4	8
Om06	*H_e_*	0.921	0.902	0.807	0.951	0.609	0.910
	*f*	0.486*	0.631*	0.660*	0.549*	0.851*	0.519*
	*N_a_*	13	11	9	16	3	19
Om09	*H_e_*	0.941	0.950	0.917	0.943	0.886	0.961
	*f*	0.420*	0.330	0.865*	0.189	0.812*	0.548*
	*N_a_*	12	13	20	16	8	19
Om12	*H_e_*	0.234	0.275	0.295	0.090	0.000	0.281
	*f*	−0.125	0.273	0.321*	−0.012	0.000	0.063
	*N_a_*	2	4	6	3	1	3
Om02	*H_e_*	0.890	0.876	0.860	0.900	0.859	0.926
	*f*	0.198	0.391*	0.240*	0.259	0.642*	0.494*
	*N_a_*	12	9	10	13	8	16
Om07	*H_e_*	0.933	-	0.852	0.950	0.833	-
	*f*	0.821	-	0.871*	0.579	0.829*	-
	*N_a_*	6	-	13	6	5	-
Om08	*H_e_*	0.219	0.050	0.025	0.085	0.401	0.118
	*f*	−0.087	0.000	−0.003	−0.023	−0.247	−0.034
	*N_a_*	3	2	3	2	3	4
Om10	*H_e_*	0.923	0.886	0.930	0.882	0.814	0.918
	*f*	0.409	0.692*	0.846*	0.284	0.693*	0.455*
	*N_a_*	10	8	23	12	7	20

*indicates significant deviation (*P*<0.05) from expected Hardy-Weinberg equilibrium conditions.

NB/- Data missing (i.e. >50% sample drop out for locus Om 7 in S Wales and W Scotland regions).

Linkage disequilibrium was not detected among loci within most regions, with just four pairs of loci showing significant (*P*<0.05, *k* = 28 or 36) linkage disequilibrium: Om02–Om06 (South England); and Om03–Om09, Om02–Om03, and Om02–Om09 (West Scotland). The exception to this was the sample from North Wales, as every locus-pair combination (except for the eight comparisons involving Om08) was in significant linkage disequilibrium (*P*>0.05, *k* = 36). This effect largely reflects the intra-site population subdivision since, when the North Wales sample was partitioned into the two distinct clusters identified by structure analysis (see below and Figure 1), just 6 and 12 locus pairs (out of the 36 possible combinations) were significantly out of linkage equilibrium; indeed, just three pairs of loci (Om06 & Om09, Om02 & Om09, Om02 & Om10) were out of linkage equilibrium in both of these sample clusters.

Bayesian analysis of population structure provided increasing model support as the number of populations (values of *K*) increased, but did not uncover a distinct modal value of *ln* P(D); in addition, it was difficult to identify an objective “plateau” to the variation in *ln*P(D) against *K* and thus no obvious partition of the data was evident using the value of *ln*P(D) itself. By contrast, *ΔK* provided clear support for a bipartition of the data set (Figure 1). Interestingly, the two hypothetical populations do not correspond with any obvious large scale geographical feature or regional boundary. Rather, the major partition in the data set occurred locally within the large sample from a single beach in North Wale. Thus, almost all (115 out of 119) of the isolates from areas outside North Wales were assigned to the same model cluster (cluster 1) with a probability of membership of greater than 0.75. Within the North Wales shore, approximately one third (29 out of 81) of the isolates had a high probability of membership (>0.75) to this same model cluster, while most (49 isolates) of the remaining samples from North Wales were assigned with a similarly high membership probability to a distinct second population (cluster 2); just 3 of the North Wales isolates apparently were admixed (Figure 1). Elsewhere, similarly there was little evidence of admixture between the two model clusters, although isolates from South England tended to have greater (but still low) probabilities of membership to the North Wales cluster 2.

**Figure 2:**
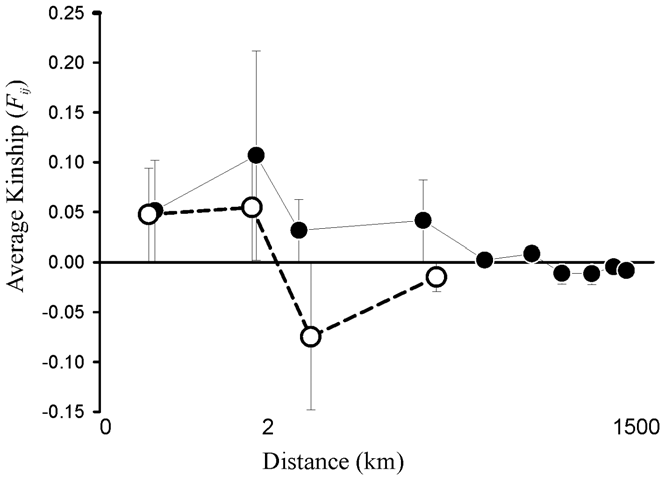
Spatial-autocorrelation-based isolation by distance plot indicating average (all loci) kinship (Fij with 95% confidence intervals, 40) as a function of distance (km) for the 2 model clusters identified in **Figure 1**. Filled and open circles corresponded to model clusters 1 and 2, respectively (following Figure 1).

Genetic differentiation between regions was weak to moderate, varying from *F_ST_* <0.01 for four regional pairs, *F_ST_*  = 0.015–0.089 between a further ten regional pairs, and *F_ST_* >0.12 only between North Wales and East Scotland (Table 3); a re-analysis of data based on the results described above identified greatest genetic differentiation (*F_ST_*  = 0.166) between the two hypothetical clusters (identified by structure) within the North Wales site. There was little evidence for an IBD structure (*i.e*. a gradual decrease in *F_ij_*) throughout the general distribution of our samples, but rather an apparent clustering of similar genotypes within shores. Thus, isolates tended to be genetically similar (average *F_ij_* variable but positve) at a small spatial scale (∼1–2 km), but thereafter average pairwise kinship among isolates (*F_ij_*) remains close to zero irrespective of the distance class (Figure 2).

**Table 3:** Pairwise estimates of *F_ST_* between samples of *O. marina* from six regions in the UK; *indicates significant difference between population pairs (*P*<0.05).

	S Wales	N Wales	W Ireland	E Scotland	W Scotland
**S England**	0.0155	0.0348*	0.0011	0.0857*	−0.0107
**S Wales**		0.0630*	0.0189*	0.0609*	0.0052
**N Wales**			0.0510*	0.1166*	0.0353*
**W Ireland**				0.0886*	0.0082
**E Scotland**					0.0583*

## Discussion

Here, we characterise a panel of polymorphic microsatellite loci that can be used to detect subtle patterns of spatial genetic structure and assess the extent of genetic diversity in the common marine protist *Oxyrrhis marina*. Our motivation was to acquire greater resolution than that afforded by the distribution of DNA sequence variation, where analyses to date have not identified spatial genetic structure in north-east Atlantic coastal waters (*cf*. [19], [23]). To this end, we uncovered substantially more genetic diversity than previously recognised and no evidence for extensive clonality. In addition, the pattern of genetic differentiation revealed a contrast in population structure at different spatial scales: (1) weak spatial structure at a regional scale (on the order of 100 s of kms) and (2) distinct genetic boundaries within a 2 km shore.

### Microsatellite characteristics

Relatively high levels of polymorphism in these *O. marina* microsatellites are consistent with variability at microsatellite loci in other dinoflagellate species. For example, between 4 and 15 alleles per locus and gene diversities (*H_e_*) as high as 0.97 were uncovered in 5 species from the genus *Alexandrium* [42], [43], [44], [45], [46], and estimates of *H_e_* vary between 0.08–0.82, 0.08–0.88, 0.21–0.68 and 0.55–0.93 in other potentially harmful dinoflagellates (*Akashiwo sanguinea* [47]; *Cochlodinium polykrikoides* [48]; *Heterocapsa circularisquama* [49]; and *Karenia mikimotoi* [50], respectively). In free-living marine-protist taxa more generally, similarly high levels of genetic diversity have been reported [13], [51], [52]. Thus extensive intraspecific variability at microsatellite loci appears to be a common feature of many free-living marine protists in contrast to relatively low levels of sequence diversity at mitochondrial and ribosomal genes.

Another feature of microsatellite loci in marine protists appears to be a high frequency of null alleles (*i.e.* presence of non-amplifying alleles) [13], [14], [15], [53]. For *O. marina*, locus Om07 displayed a high frequency of non-amplifying samples, contributing to potentially high error rate without repeat genotyping, and another locus (Om06) showed a strong tendency toward heterozygote deficiency. Presumably, high frequencies of null alleles are a result of mutations in flanking sequence, but whether they indicate an elevated rate of mutation within nominal species or reflect the occurrence of cryptic species is unclear. High levels of cryptic genetic diversity certainly occur within *O. marina*, with present analysis of globally-distributed isolates culminating in two genetically divergent lineages (*i* and *ii*) that each consist of two distinct clades [54]. Our partial genomic library was constructed using DNA from isolates belonging to *O. marina* lineage *i*, which so far has been found to dominate samples from northern European coastal waters [20]. PCR tests demonstrate that primer pairs fail to amplify PCR products in isolates from lineage *ii* (data not shown), which has been proposed to be a separate species (designated *Oxyrrhis maritima* [54]); these data thus highlight the potential pitfalls for researchers wanting to develop microsatellite loci in relatively understudied protist morphospecies that may harbour high levels of genomic variation or even represent cryptic species.

### Spatial genetic structure

Patterns of sequence variation among populations of free-living marine protists have been interpreted both in terms of processes that may lead to genetic homogeneity [55], [56] or drive genetic divergence among regions [19], [53]. Such studies, however, typically encompass large spatial scales (*i.e*. across ocean basins or even between poles) and tend to overlook patterns of variation within localities. This sampling strategy probably reflects a commonly, though not universally (see Introduction) held view that local populations of free-living protists should be unstructured as a consequence of large population sizes and the potential for wide dispersal [1], [2], [58].

The recent application of highly polymorphic genetic markers (such as microsatellites), in combination with intensive sampling strategies, have provided greater resolution to determine fine-scale spatial structure in populations of marine protists. In some cases studies have supported a lack of population differentiation; for example, microsatellite-based studies on several diatom species have failed to uncover significant genetic divergence among samples within regional seas [52], [59]. Conversely, there are increasing numbers of examples of distinct population structure occurring on small spatial scales in a range of free-living protists [9], [11], [53], [60]. Thus two issues are becoming clear: firstly, broad generalisations and assumptions about the characteristics of free-living protist distributions are unlikely to be helpful; secondly, studies of population structure in these organisms almost certainly require the application of highly polymorphic genetic markers, as substantial genetic diversity is potentially overlooked when fewer, less variable (*i.e*. sequence) markers are used. A comparison of genotype data for *O. marina* from the study presented here reinforces the above points. That we identified more than 180 unique genotypes within our sample of 200 *O. marina* isolates from the coastal waters of Great Britain and Ireland is an obvious contrast to the single haplotype indentified across the same region based on sequence variation at COX1 and 5.8S-ITS [19].

Notwithstanding the localised genetic differentiation within the sample from North Wales (see *Fine-scale divergence*, below) individual model-based clustering did not uncover substantial spatial genetic structure across much of the coastal waters of Great Britain and Ireland (*cf*. Figure 1) and there was no pattern of IBD throughout the region. Other population-genetic studies on marine species along the western coast of Great Britain similarly have failed to uncover substantial spatial genetic structure where species have a planktonic dispersal phase [61], although in some mobile species subtle IBD has been identified (e.g. plaice, [62]). More generally, substantial spatial genetic structure around the UK occurs among populations of sessile or sedentary invertebrates that lack a planktotrophic larva [61], [63]. Nonetheless, low levels of genetic divergence among regional locations did occur (nearly half of the pairwise estimates of *F_ST_* were greater than 0.05), indicating that *O. marina* is not panmictic at this scale. Such levels of genetic differentiation are relatively low compared to other dinoflagellates (*e.g*. *C. polykrikoides* in the Sea of Japan; [11]). Similar to the study by Nagai and co-workers, our analysis identified few model clusters/nominal populations and did not reveal the underlying pattern of spatial structure to be driven simply by the distance separating populations. In a more general context the emerging picture for populations of free-living marine protists is one of complex patterns of regional population divergence that likely reflect the combined influences of, for example, current patterns, environmental conditions, historical contingency and anthropogenic transportation [5], [11], [4], [57]. A better understanding of the relative roles of such potential drivers of spatial structure requires further sampling, which for *O. marina* is ongoing in our research group. At present, this work highlights our lack of knowledge about dispersal by microbial species, as there remain only a handful of genetic studies conducted at the appropriate resolution to assess population-level processes. Illustratively, free-living protists are notably absent from reviews of dispersal scale and genetic differentiation exhibited by marine taxa, despite their ecological importance [64], [65].

### Fine-scale divergence

Two major features are apparent from the fine-scale analysis of samples from a single shore: (1) genotypic diversity at a local scale is high and (2) a conspicuous population division occurs at this location. Contrary to our expectation that one or few clonal genotypes may dominate, particularly in the 81 isolates collected from the 2 km stretch of shore in North Wales, we identified a large number of unique genotypes, as indeed have the few studies of other free-living protists that have genotyped many samples from the same location [15], [52]. Whilst outside of the scope of the current study, it will clearly be important to determine the temporal stability of such patterns, particularly as short generation times (on the order of hours to days for many *O. marina* [24]) and typically pronounced patterns of seasonal abundance [24] potentially serve to rapidly modify population structures. Further, extensive genotypic diversity and a lack of strong linkage between loci certainly infer the occurrence of sex and recombination [66]. While sex has been documented in this species [24], we have a limited understanding of life history of *O. marina* in natural populations and no estimates of the frequency or periodicity of sex. Clearly estimates of the rate of genetic recombination are fundamental for understanding population genetic structure, thus such estimates remain a priority for future study.

More generally, these observations raise important questions about the possible mechanisms that sustain the apparently high levels of diversity in populations of free-living protists. For example, large population sizes potentially harbour high levels of neutral genetic diversity simply as a function of effective population size and largely irrespective of the underlying selective regime [67]. Alternatively, high genetic diversity may reflect adaptive variation - certainly many studies have reported substantial ecophysiological variation among clonal isolates of protists [68], [69], including *O. marina* [23]. Whether such physiological variation might influence coexistence between genotypes or reflect ecological partitioning at local scales has not yet been assessed. Finally, while genotypic diversity within populations of active-cells may be ephemeral, long term diversity may be maintained via cyst formation and the presence of extensive ‘cyst banks’ typically within aquatic sediments. Again, though cyst formation is well documented for a broad range of protist taxa [58], and tentatively for *O. marina*, [70]), the actual extent of the strategy, and its role in shaping population demography, has not been extensively assessed (though see [71] for an example). Encouragingly, the recent bloom in development of microsatellites loci for marine protists should facilitate a better understanding of seasonal- and long-term population dynamics, particularly at fine spatial scales (see also [15], [72], [73]). While we remain largely ignorant of such mechanisms, and an assessment of their relative contribution to shaping patterns of genetic diversity in free-living protists remains a subject for future study, these data highlight an important and pragmatic need for researchers to characterise their experimental strains to provide much needed context to any comparisons between isolates [54]. Indeed, for *O. marina*, which is a commonly employed ecological-model organism [22] high levels of diversity potentially provide an opportunity to assess the impact of intraspecific diversity on a range of ecological processes.

The partition of the sample from North Wales into two model populations, one of which was common with other UK regions, and the other of which was unique to this location, was unexpected. The occurrence of a unique, highly divergent population at this single site is certainly curious. It is possible that this pattern reflects the greater sampling effort at the North Wales site and the concomitant statistical power to resolve population structure (*i.e*. ability to model linkage disequilibrium and Hardy-Weinberg equilibrium conditions) at this location as compared with elsewhere (see [36] for methodological details). However, we think this unlikely, as the sample sizes and marker polymorphisms are sufficient to detect additional populations and similar within-sample patterns are evident in comparable analyses of, for example, *C. polykrikoides* [11]. It is unlikely that this additional population represents the presence of cryptic species of *Oxyrrhis*, since both mitochondrial and nuclear sequence variation between these isolates is absent [19]. Indeed, the presence of individuals with an apparent mixed ancestry (Figure 1) suggests some degree of genetic exchange between the model clusters. In addition, using spatial autocorrelation we detected some clustering of genetically similar isolates at fine scales (<2 km) within both model clusters that, given the above discussion, cannot be due to local clonal abundance. The issue of how genetic differentiation is maintained on such a small spatial is also unclear. *Oxyrrhis marina* has been documented to form ephemeral adherent cysts associated with the tidal cycle [70]; whether dormant/resting cysts are also produced and whether long term dormancy could provide a mechanism for partial genetic isolation are subjects of ongoing research in our laboratory.

To conclude, these new microsatellite loci have uncovered spatial structure at a greater resolution than afforded by sequence markers commonly-employed for phylogeographic analysis of free-living marine protists. *Oxyrrhis marina* is not panmictic at spatial scales between 2 and ∼1000 km within Great Britain and Ireland. These data highlight extensive levels of genetic diversity, but raise questions about the mechanisms that maintain such variation. Studies, such as the one reported here, employing population genetic approaches at a range of spatial scales, are increasingly providing the molecular tools to estimate demographic parameters and explore the adaptive mechanisms behind spatial and seasonal variation in a broad range of free-living protists.
